# Supplementary figures and images for: High resolution DLP stereolithography to fabricate biocompatible hydroxyapatite structures that support osteogenesis
Source: PLoS One. 2022 Aug 8;17(8):e0272283. doi: 10.1371/journal.pone.0272283 (PMC9359536; doi:10.1371/journal.pone.0272283)

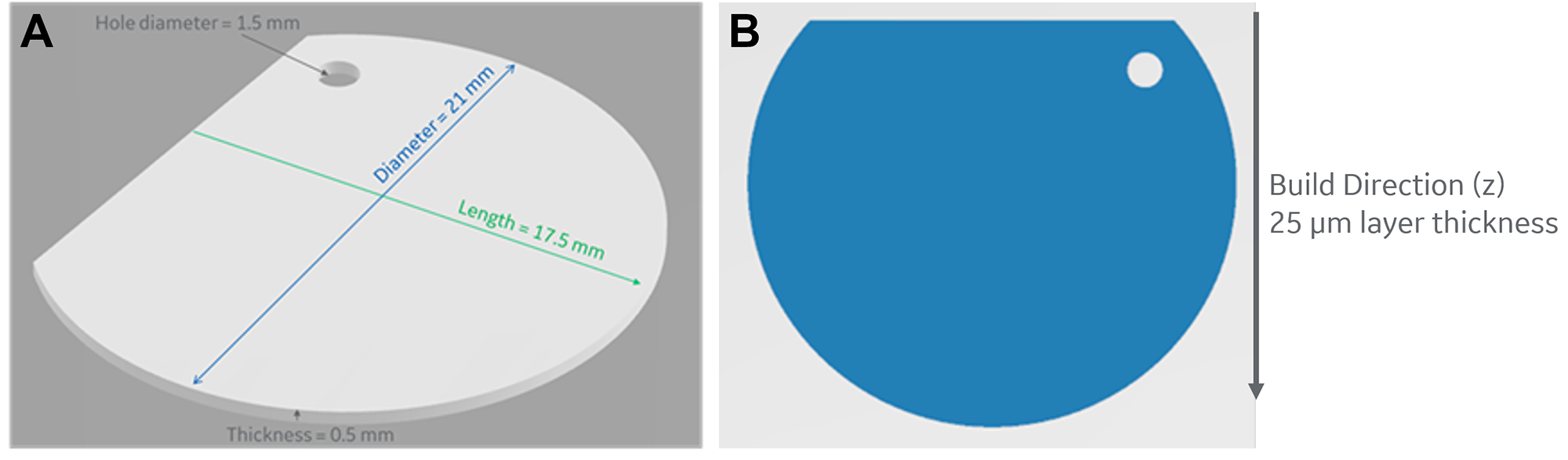

Supplement: S1 Fig — (A) Geometry of the build was a circular disc with a hole on the edge to facilitate handling with sterile forceps. (B) The flat bottom is adhered to the build plate and the layers are built up in the direction of the arrow. (TIF) [file pone.0272283.s001.tif]

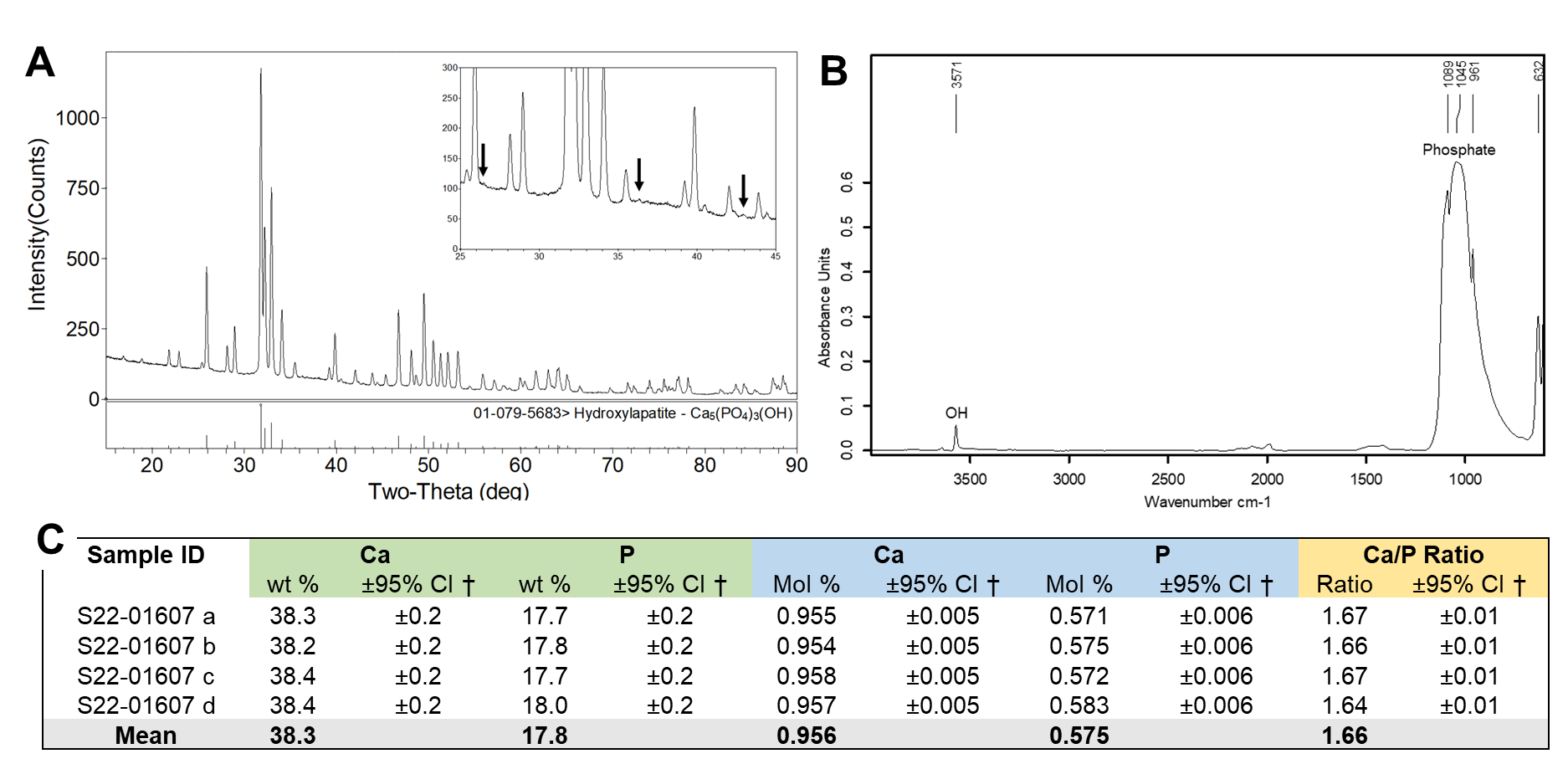

Supplement: S2 Fig — (A) The fired coupon was analyzed by X-ray diffraction (XRD) to evaluate the crystalline phases. The XRD pattern was consistent with hydroxyapatite and showed only three very minor secondary phase peaks, which were consistent with orthorhombic hydroxyapatite and magnesium oxide (arrows in inset). (B) The coupon was also analyzed by Fourier-transform Infrared Spectroscopy (FTIR), revealing the IR spectrum of LithaBone HA400 powder in diamond transmission cell. (C) Inductively Coupled Plasma-Optical Emission Spectroscopy (ICP-OES) was used to characterize the average calcium/phosphate (Ca/P) ratio of coupons. Analysis was done on fired (sintered) LithaBone HA400 powder (Sample IDs S22-01607a-d). Wt%, Mol%, and Ca/P ratio was measured. (TIF) [file pone.0272283.s002.tif]

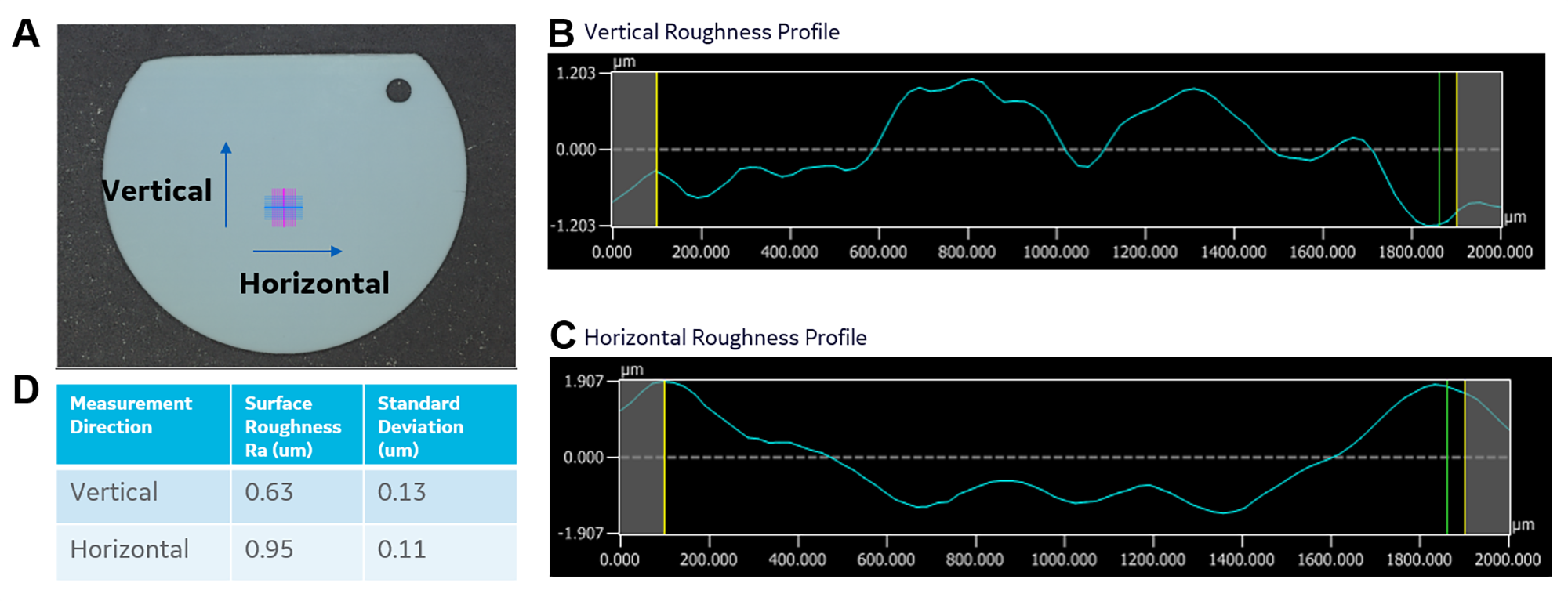

Supplement: S3 Fig — (A) Image of coupon at 12x magnification showing location of five 2 mm parallel lines in vertical and horizontal directions. Example multi-line roughness profiles in the (B) vertical and (C) horizontal directions with (D) values displayed in the table. (TIF) [file pone.0272283.s003.tif]

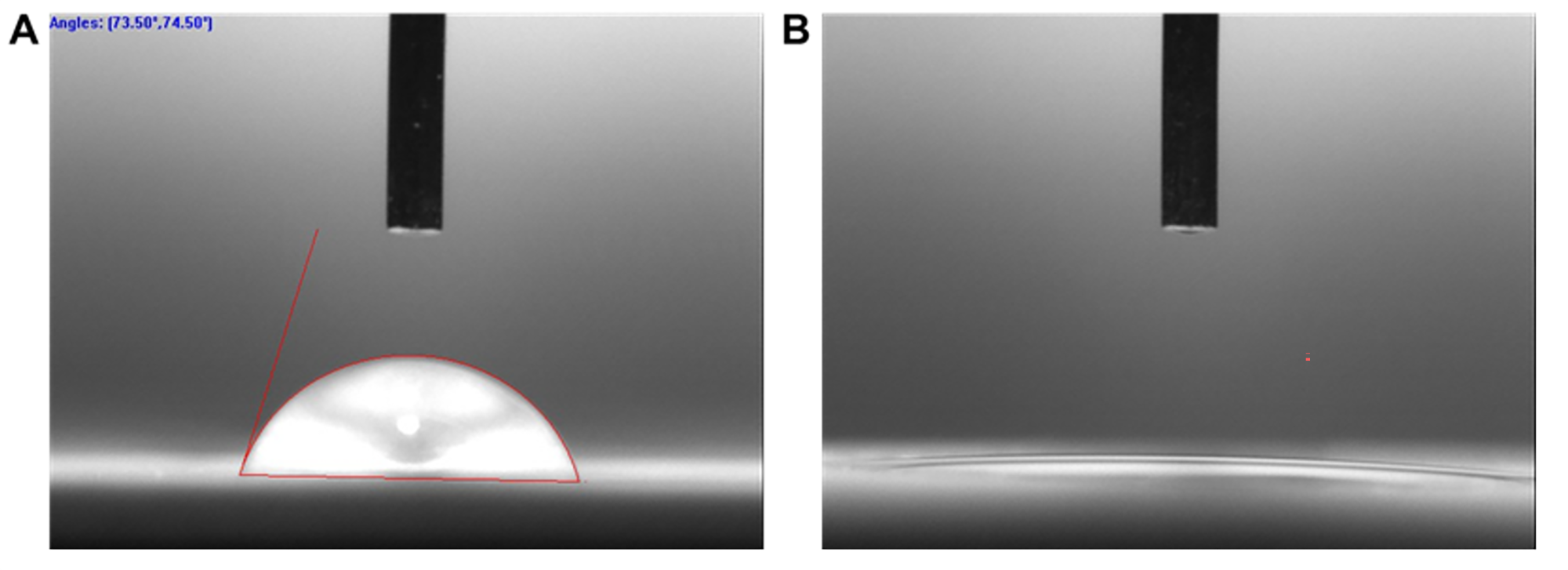

Supplement: S4 Fig — (A) Pictures of water droplet on coupon surface, image taken 2 seconds after liquid contacted the surface of the coupon, resulting in a contact angle of (L,R) 73.5°, 74.5° and (B) a contact angle of (L,R) 0°, 0° with the water fully spread across coupon surface. Images demonstrate the contact angle approach used to measure hydrophilicity of coupons. (TIF) [file pone.0272283.s004.tif]

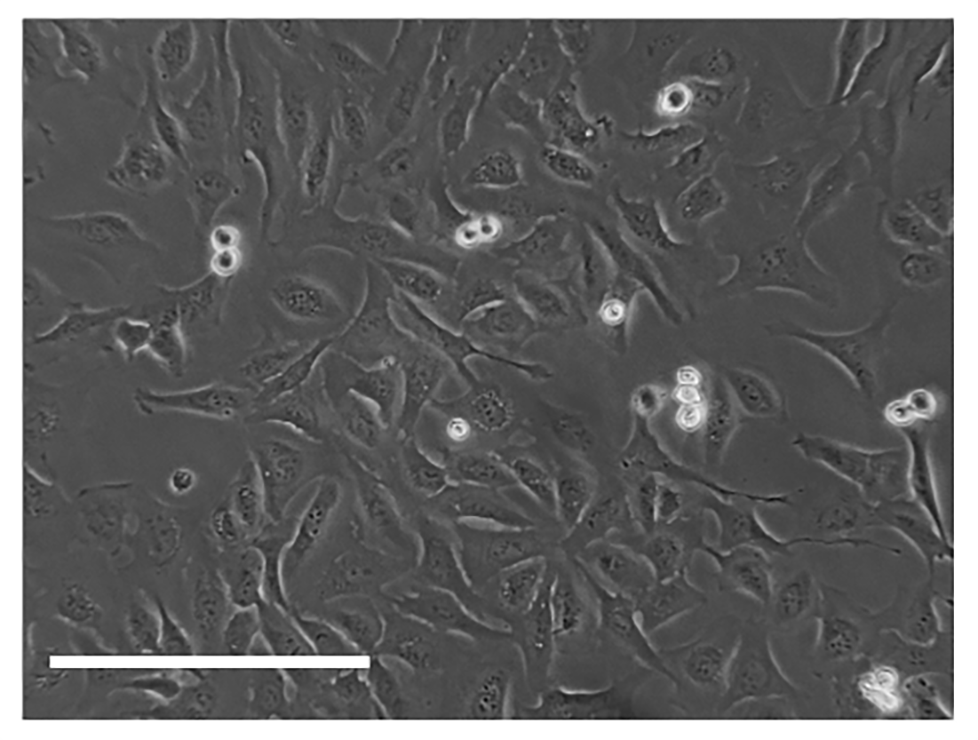

Supplement: S5 Fig — Seeded at ~66% confluency (4.6x10^3^ cells/cm2), U2OS cells were exposed to coupon conditioned culture media for 48 hours. Cells cultured on tissue culture polystyrene (TCP) and in the presence of coupon conditioned culture media were observed to maintain high confluency with no negative impact on morphology or proliferative capabilities. Image was taken using EVOS, phase contrast, 10x objective magnification, scale bar is 250μm. (TIF) [file pone.0272283.s005.tif]

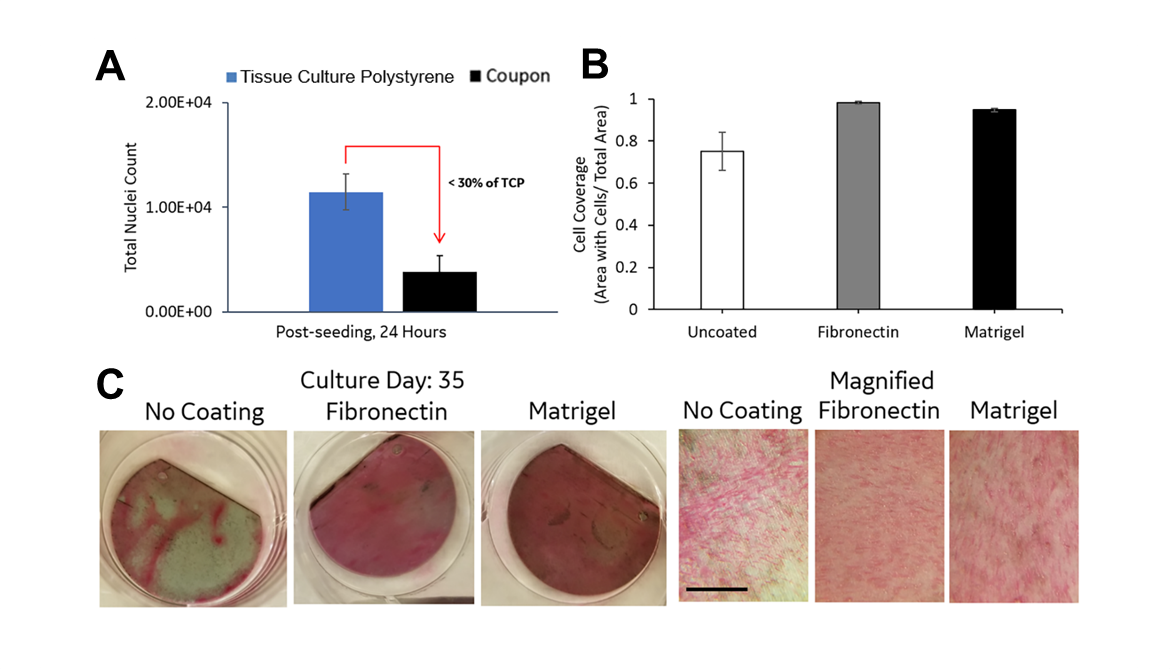

Supplement: S6 Fig — (A) MSCs were seeded at a targeted density of ~25% (6.25x10^3^ cells/cm2) onto tissue culture polystyrene (12-well plates, “TCP”) and coupons. Image analysis was used to measure total number of nuclei 24 hours after seeding for a surface area of ~3cm2. Coupons have ~30% fewer number of cells compared to tissue culture polystyrene, “TCP”. (B) Cell coverage was measured using image analysis (by image J) of uncoated HA coupons and coated coupons (fibronectin and Matrigel). Cell coverage was measured as the area covered by cells divided by total area (n = 4). (C) MSCs cultured for 35 days on HA coupons coated with fibronectin or Matrigel had greater confluency (red and pink stained region are cells, cultured in standard MSC culture media). Uncoated HA coupons had lower percent cell coverage compared to coated HA coupons (fibronectin or Matrigel, collagen). Coating HA coupons-maintained confluency of nearly 100% for up to the 35th day of culture. Cells were stained with nuclear fast red and have a pink/fuchsia color. Scale bar 1.25mm, Leica dissection microscope was used to image surfaces of HA coupons at 4x objective magnification (magnified images). (TIF) [file pone.0272283.s006.tif]

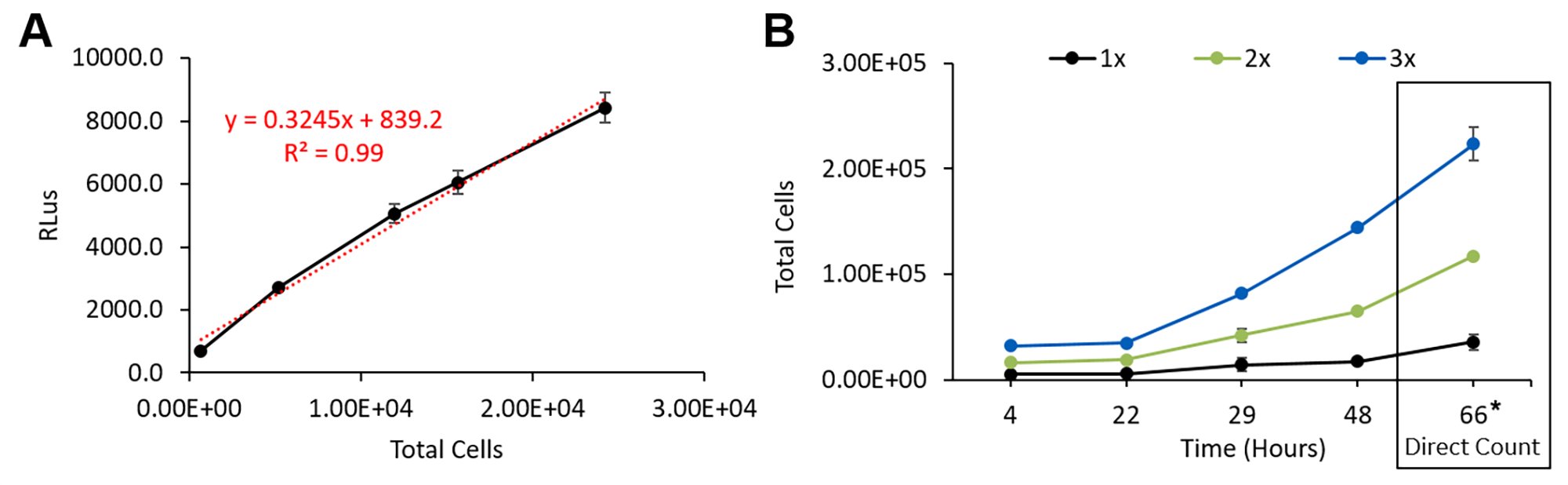

Supplement: S7 Fig — (A) RealTime-GloTM MT Cell Viability Assay was used to measure relative luminescence (RLUs) of U2OS cells seeded on standard tissue culture polystyrene (12-well plates) at cell densities to calculate a linear equation correlating RLUs with total cells. (B) To analyze accuracy of linear equation and determine functionality of the RealTime-GloTM MT Cell Viability Assay, U2OS cells were seeded at different target densities starting ~25% (1x) and increasing by a factor of 2x and 3x. Total number of cells were indirectly measured (calculated based on RLU measurements) for incubation time points 4, 22, 29, and 48 hours and directly (using image analysis) for time point 66 hours. (TIF) [file pone.0272283.s007.tif]

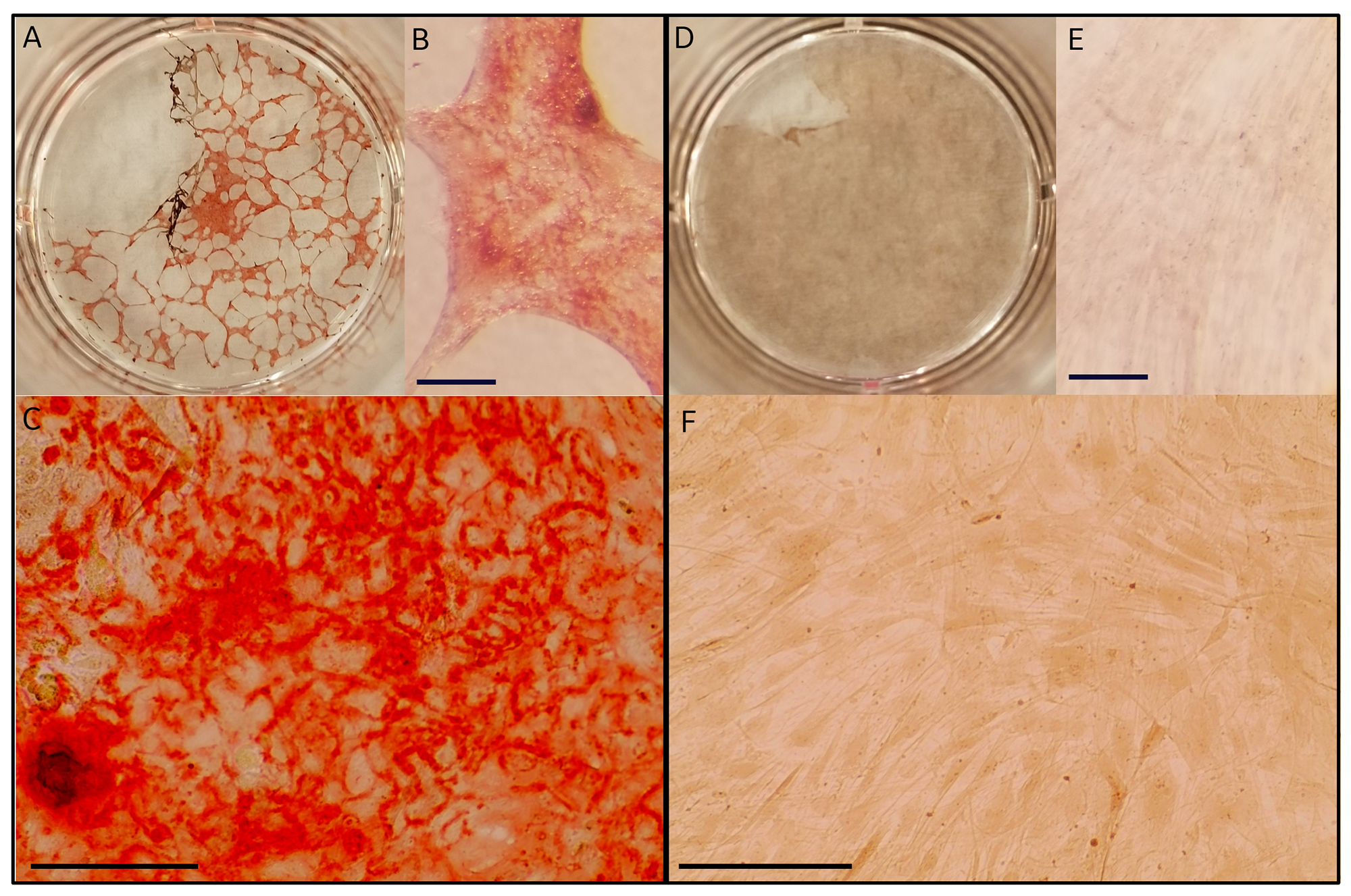

Supplement: S8 Fig — (A-C) MSCs were cultured for 14 days in either osteogenic differentiation media or (D-F) non-differentiation media. On day 14, cells were stained with Alizarin Red S dye. (B and E) Images taken at 4x objective magnification and (C and F) at 20x objective magnification. (B and E) Scale bar is 1.25mm and (C and F) 0.25mm. (TIF) [file pone.0272283.s008.tif]

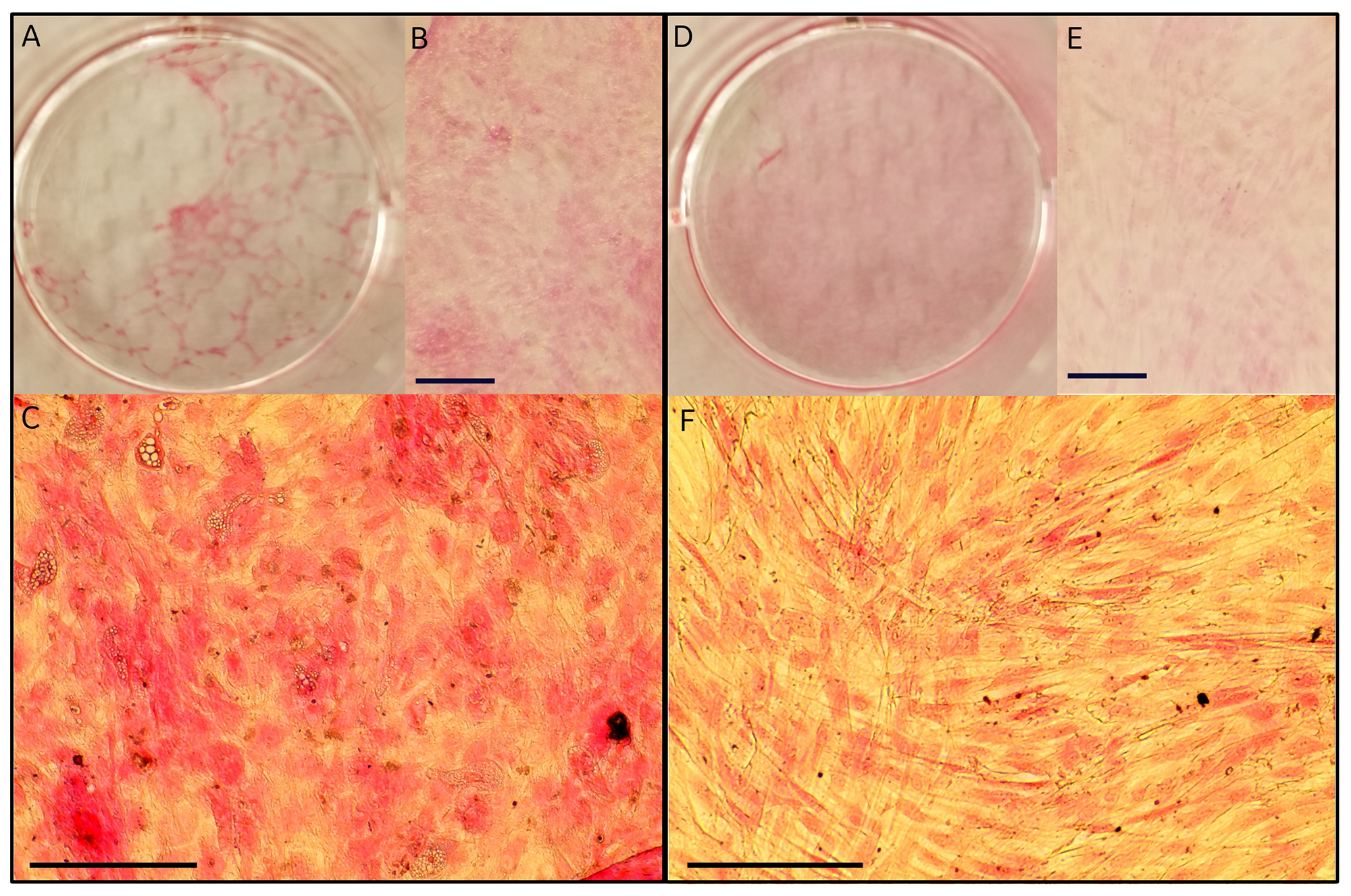

Supplement: S9 Fig — (A-C) MSCs were cultured for 35 days in either osteogenic differentiation media or (D-F) non-differentiation media. On day 35, cells were von Kossa stained. (B and E) Images taken at 4x objective magnification and (C and F) at 20x objective magnification. (B and E) Scale bar is 1.25mm and (C and F) 0.25mm. (TIF) [file pone.0272283.s009.tif]

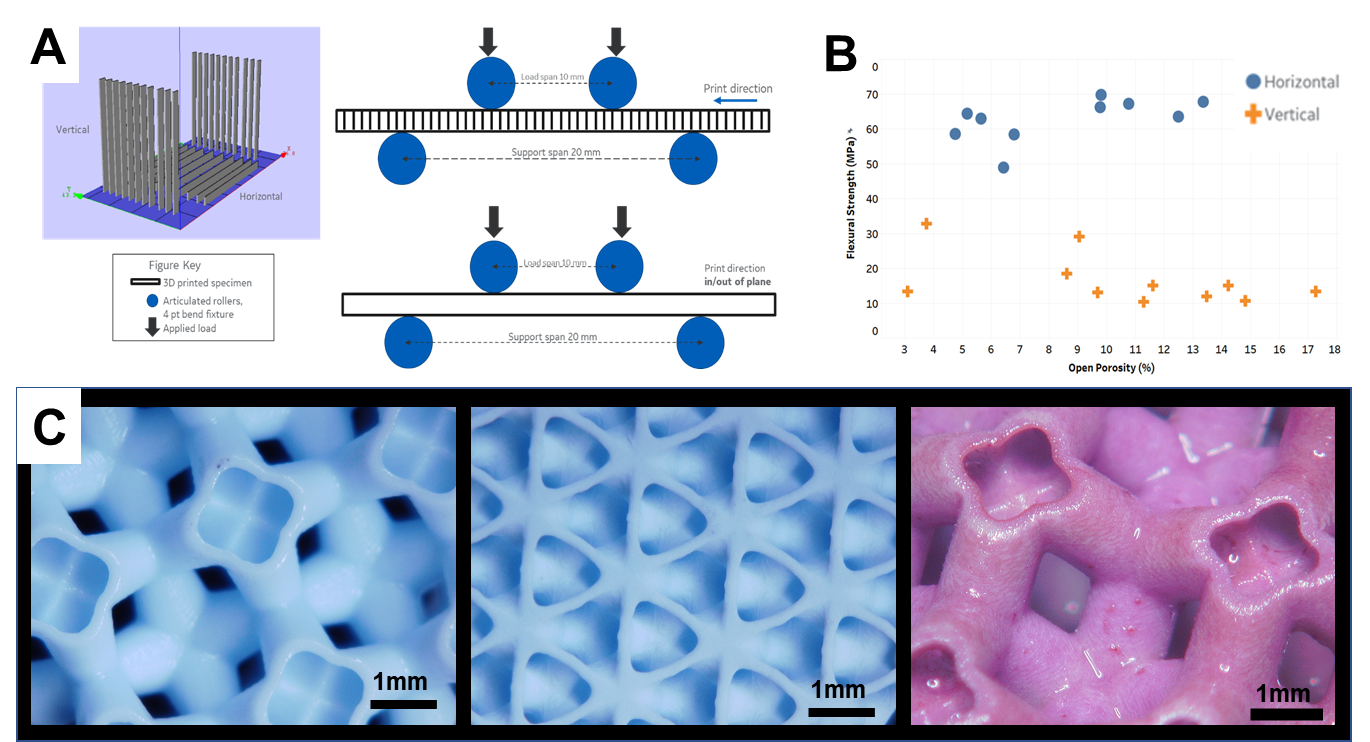

Supplement: S10 Fig — (A) Illustrations of an example build plate for DLP 3D printing of LithaBone HA400 horizontal and vertical rupture test bars for a four-point bend flexural test set up, where flexural strength is measured for vertical (top) and horizontal (bottom) 3D printed bars. (B) Plot of flexural strength (MPa) versus open porosity (%) for fired (sintered) LithaBone HA400 modulus of rupture test bars, depend on their print orientation (horizontal vs vertical). (C) Clover and trifurcating LithaBone HA400 scaffold designs for proposed use in bone formation experimental studies. Images of scaffold were taken at 200x magnification using a Hirox 3D digital microscope and right most image is of a clover design LithaBone HA400 scaffold stained with nuclear fast (pink/red; this stain is part of the von Kossa staining protocol and it stains nuclei) to visualize presence of U2OS cells after 14 days of culture. Scale bar is 1mm. (TIF) [file pone.0272283.s010.tif]
