# Supplementary material for: High resolution DLP stereolithography to fabricate biocompatible hydroxyapatite structures that support osteogenesis
Source: PLoS One. 2022 Aug 8;17(8):e0272283. doi: 10.1371/journal.pone.0272283 (PMC9359536; doi:10.1371/journal.pone.0272283)
Supplement: Attachment — Submitted filename: Jessica Martinez_Response-to-Reviewers.docx [file pone.0272283.s011.docx]

Aldo Boccaccini

Academic Editor

*PLOS ONE*

April 20, 2022

Dr. Aldo Boccaccini and PLOS ONE editorial staff:

In response to academic editor and reviewer responses regarding our original manuscript (*PONE-D-21-34494*) entitled “High resolution DLP stereolithography to fabricate biocompatible hydroxyapatite structures that support osteogenesis”, originally by Jessica S. Martinez, Sara Peterson, Cathleen A. Hoel, Daniel J. Erno, Tony Murray, Linda Boyd, Jae-Hyuk Her, Fiona Ginty, Steven J. Duclos, Brian M. Davis, and Gautam Parthasarathy, has been revised. We have outlined each point of concern and outlined how each was addressed in our revised manuscript.

1.) Two new authors were added to the revised manuscript. These new authors, **Nathan Mclean** and **Robert David**, both significantly contributed toward the material analysis studies of our 3D printed coupons. In the cover letter we identify this change to our revised manuscript and subsequent statements regarding our Funding Statement.
2.) *“…in the Acknowledgments Section of your manuscript…We note that you have provided funding information that is not currently declared in your Funding Statement…Please remove any funding-related text from the manuscript…”*

**Correction:** In the Acknowledgment Section we made suggested changes and removed statements regarding funding. In the cover letter we also addressed changes that need to be made to our Funding Statement.

Changes to Funding Statement (see Cover letter):

*This work was funded by a research grant from GE Research. The authors Jessica S. Martinez, Sara Peterson, Cathleen A. Hoel, Daniel J. Erno, Tony Murray, Linda Boyd, Jae-Hyuk Her, Nathan Mclean, Robert Davis, Fiona Ginty, Steven J. Duclos, Brian M. Davis, and Gautam Parthasarathy contributed to the design of the studies. Authors Jessica S. Martinez, Sara Peterson, Cathleen A. Hoel, Daniel J. Erno, Steven J. Duclos, Brian M. Davis, and Gautam Parthasarathy co-wrote the manuscript. The funders had no role in study design, data collection and analysis, decision to publish, or preparation of the manuscript.*

Changes to Acknowledgment Section (lines 559-564):

*We would like to thank the General Electric Company and colleagues at GE Research, particularly Kenneth Conway for consultation on best practices for sterilization and Liz McDonough in the Bioscience organization for her extensive help in using the Cell Dive imaging platform.*

3.) *“…PLOS requires an ORCID iD for the corresponding author in Editorial Manager on papers submitted after December 6th, 2016...”*

**Correction:** Corresponding author, Jessica S. Martinez, has updated her ORCID iD in Editorial Manager for PLOS ONE.

4.) “…We note that you have included the phrase “data not shown” in your manuscript. Unfortunately, this does not meet our data sharing requirements…”

**Correction:** The information discussed in lines 521-525 of the original manuscript was not a core part of the research and rather an observation, which was not investigated with statistical and rigorous analysis. It was an observation from the authors of the already known donor-to-donor variability in human mesenchymal stem cell lines. As such, we have removed the phrase and have made the following changes to the revised manuscript, including a reference to known donor variance in MSC cell lines:

(Lines 624-628) *After 35 days of culture, in both native tissue culture and HA coupons, the MSCs contracted leaving areas along the surface free of cells with the degree of contraction varying between donor MSC cell lines purchased from Lonza (MSC cell line donor variance were expected [56]), but the level of contraction was not notably different between tissue culture plastic controls and HA.*

Reference added:

*56.) Trivedi A, Miyazawa B, Gibb S, Valanoski K, Vivona L, Lin M, et al. Bone marrow donor selection and characterization of MSCs is critical for pre-clinical and clinical cell dose production. Journal of Translational Medicine. 2019;17: 128. doi:10.1186/s12967-019-1877-4*

5.) Reviewer 1 minor comment. “…In the section “Materials and methods” the author make no references to the figures, these references are made later in the section about “Results”. This sometimes makes it difficult to understand certain aspects in the text. I suggest to reference the relevant figures (e.g. sketch of working principle, geometry of coupons, …) also in the beginning of the text.

**Correction:** Reviewer 1 brings up a relevant point regarding the need for figure references in the Materials and Methods section to describe how our 3D printed materials were created and analyzed. To address this concern, we have made the following changes to the revised manuscript:

(Lines 118-123) *Using the DLP printing system (outlined in Fig 1), a modified circular coupon geometry (17.5mm length, 21mm diameter, and 0.5mm thickness) (see Fig 2, A for image of coupon after printing and S1 Fig for illustrations of coupon dimensions and print direction) was selected to maximize the coverage area of a single cell in a standard 12 well plate and provide feasibility for material characterization and biocompatibility.*

(Lines 132-148) *Commercial LithaBone HA400… Nominal dimensions of coupons were 17.5 mm tall, 21 mm diameter, and 0.5 mm thick, see S1 Fig.*

(Lines 160-161) *The coupons were light blue in color, consistent with vendor data sheet, see Fig 2, A.*

(Lines 177-193) *The data was collected with the VANTEC-500 2D area detector for 30 minutes for each coupon…* ICP-OES analysis results for Ca, P, and Ca/P ratios are shown in S2 Fig.

(Lines 207-216) *Samples were processed and roughness analyzed using the standard Keyence software for the profilometer, see S3 Fig. SEM images…see Fig 2, C. Contact angle was measured…see S4 Fig.*

6.) Reviewer 1 major comment: “…I am missing some numbers on mechanical properties of the used materials, since the strength of the used HAP determines its usability in the clinical setup. Values on bending or biaxial bending strength would certainly add a lot of value to the paper.”

**Correction:** Although reviewer 1 concerns regarding the strength of the commercial LithaBone HA400 slurries used after sintering are important, we feel these analyses are beyond the scope of this manuscript, which address the biocompatibility of DLP printed materials with human cell lines (i.e. MSCs). As such, we did not perform bending or biaxial bending strengths analysis on these circular coupons. However, to reviewer’s 1 concern, we plan to address this point in a more in-depth, future study, where we will expand our scope of work to measure and interrogate geometry dependent changes to the inherent strength of the LithaBone HA400 material. To clarify reviewer 1 point of concern, we have included more information regarding the composition and characteristic of the LithaBone HA400 material as documented by the commercial company we purchased the slurries from. In addition, we have also made the following changes/comment in the revised manuscript to reflex these forward-looking efforts regarding material strength analysis:

(Lines 138-141) *According to Lithoz’s technical data, the LithaBone HA400 slurry has a solid loading (vol%) of 46 and a dynamic viscosity (Pa-s at 50 s^-1^)of 5-10 and sintered ceramic from these slurries are expected to have a Theoretical density (g/cm^3^) of 3.**16, a Relative density (%) of 85, and final fired composition of hydroxyapatite (HA).*

(Lines 646-667) *The 3D scaffold structures will be designed to support cell growth, vascularization and bone formation, while pushing towards mechanical properties, including compression and rigidity, comparable to bone. Using a four-point bend flexural study and rupture test bars with dimensions that differ from ASTM testing standard C1161, we were able to measure flexural strength of sintered LithaBone HA400 prints, see S10 Fig. Not surprisingly, the inherent strength of LithaBone HA400 is lower than that of human cortical bone. As shown in S10 Fig, A, decreased vertical strength was expected due to build layer orientation in the four-point bend test setup. Decreased strength due to build layers sitting parallel to the fracture axis (vertical) as opposed to perpendicular (horizontal) has been shown by anisotropic mechanical behavior of ceramic additive components and is well reported in the field of additive manufacturing [57]. Material defects (e.g. build layer cracking and interlaminar porosity) can disproportionally impact the flexural strength measurement in the vertical direction. Both print orientation and geometry are variables for controlling and optimizing the mechanical properties of additively printed materials [58], including LithaBone HA400 prints [59,60], and should be further investigated. In addition, changes to inherent strength of LithaBone HA400 dependent on scaffold geometry should be analyzed, with an in-depth investigation into its potential usability in both loading-bearing and non-loading bearing orthopedic applications. DLP constructed scaffolds should be evaluated for mechanical properties including its strength, compression, bending and biaxial bending, which will provide relative insight into its potential application for clinical use.*

**Correction-continued:** To further address reviewer 1 concern, we included flexural strength measurement from our flexural strength study using DLP 3D printed rupture test bars from LithaBone HA400. We have added S10 Fig, which illustrates how test bars were printed and how our four-point bend flexural test was set up. We have updated the Material and Methods section to include detailed description of this method and have discussed measured flexural strength in the Results and Conclusion/Discussion section of the revised manuscript. As such, we have made the following changes to the revised manuscript:

(Line 165) *Phase identification, density, and characterization.*

(Lines 217-243) *Flexural strength measurements of sintered DLP 3D printed LithaBone HA400 modulus of rupture (MOR) test bars were conducted using a four-point bend flexural set-up. As depicted in S10 Fig, MOR bars (L x w x d: 40 mm x 3 mm x 0.5 mm as-printed) were printed in both the vertical and horizontal direction. An example build plate setup for printing test bars is shown in S10 Fig, A. Bars were printed on the same build plate, cleaned in the same solvent bath (blend of 55% propylene carbonate and 45% tripropylene glycol), and fired in the same furnace to isolate the effect of print orientation on sintered LithaBone HA400 fired strength. It’s important to note that dimensions of MOR test bars used differ from ASTM testing standard C1161 because thicker bars printed from LithaBone HA400 crack during firing. To avoid this, the width of our test bars was increased, providing more area under the applied load to compensate for the thin depth. The four-point bend flexural testing set-up used in these efforts is illustrated in S10 Fig, A.*

*Flexural strength was measured using a four-point bend test fixture with articulating rollers per ASTM C1161. The load frame was an Instron 4465 series with electro-mechanical 2 ball-screw frame and was operated using Instron WaveMatrix Series IX Software. Instrumentation set up was as follows: load span of 10 mm, support span of 20 mm, and displacement ramp rate of 0.01 in/min. Compression was applied via the rollers until the sample fractured and the maximum flexural strength was calculated using the equation below where F is the force in Newtons at cracking, L is the support span, w is the width of the test bar in mm, and d is the depth of the test bar in mm.*

*Max Flexural Strength (MPa) =* $\frac{0.75 \cdot F\cdot L}{w\cdot d^{2}}$

*DLP scaffold designs (clover and trifurcating) from LithaBone HA400, proposed for bone formation studies (see S10 Fig, C) were imaged using a Hirox 3D digital microscope. Scaffolds seeded with U2OS cells (stained with nuclear fast, used in von Kossa staining protocol) were also imaged.*

(Lines 360-376) *Measured open porosity values across the coupons ranged from 0.0% to 9.6%, showing a relatively broad range of final open porosity values. The variation in open porosity was expected and potentially resulting from thermal gradients in the tube furnace and the material’s sensitivity to the maximum sintering temperature. Controlling thermal gradients during sintering may improve the uniform distribution of material porosity and slurries can be engineered to produce sintered prints with target porosities [37].* *In addition, a four-point bend flexural test study was conducted, to probe at the inherent strength of LithaBone HA400 prints. Using MOR test bars with dimensions that differ from ASTM testing standard C1161 (see S10 Fig), the printing direction had a direct impact on flexural strength. Not surprisingly, the inherent material strength of LithaBone HA400 (~63±6MPa, N=10, horizontal flexural strength and ~16±7MPa, N=7, vertical strength) was lower than reported ranges for cortical bone (100-150MPa) [38]. However, a more in-depth investigation assessing impact of LithaBone HA400 print geometry on its strength, particularly for scaffolds whose structure has been optimized for load-bearing applications, would be most informative regarding the potential use of LithaBone HA 400 in bone formation.*

Reference added:

*38.) Mohammadkhah A, Day DE. Mechanical properties of bioactive glass/polymer composite scaffolds for repairing load bearing bones. International Journal of Applied Glass Science. 2018;9: 188–197. doi:10.1111/ijag.12339*

(Lines 648-678) *Using a four-point bend flexural study and rupture test bars with dimensions that differ from ASTM testing standard C1161, we were able to measure flexural strength of sintered LithaBone HA400 prints, see S10 Fig. Not surprisingly, the inherent strength of LithaBone HA400 is lower than that of human cortical bone. As shown in S10 Fig, A, decreased vertical strength was expected due to build layer orientation in the four-point bend test setup.* *Decreased strength due to build layers sitting parallel to the fracture axis (vertical) as opposed to perpendicular (horizontal) has been shown by anisotropic mechanical behavior of ceramic additive components and is well reported in the field of additive manufacturing [57]. Material defects (e.g. build layer cracking and interlaminar porosity) can disproportionally impact the flexural strength measurement in the vertical direction. Both print orientation and geometry are variables for controlling and optimizing the mechanical properties of additively printed materials [58], including LithaBone HA400 prints [59,60], and should be further investigated. In addition, changes to inherent strength of LithaBone HA400 dependent on scaffold geometry should be analyzed, with an in-depth investigation into its potential usability in both loading-bearing and non-loading bearing orthopedic applications. DLP constructed scaffolds should be evaluated for mechanical properties including its strength, compression, bending and biaxial bending, which will provide relative insight into its potential application for clinical use. In this study, a commercial slurry from Lithoz was used, however, future work should also consider integration of other slurry components (biphasic material systems) to improve the materials osseointegration.* *Such efforts might include the integration of β-tricalcium phosphate and other resorbable elements into the slurry system [18]. These types of composite slurries would have base components with both rapid (β-tricalcium phosphate) and slower (HA) rates of resorption tailored toward supporting patient specific bone repair needs. Development of novel slurries for DLP printed materials for orthopedic and clinical applications would benefit from an in-depth material characterization analysis (i.e. FTIR spectrum, particle size distribution within slurries, Ca/P ratio and degree of carbonation sintered prints) to aid in apprehension of cell response to DLP produced ceramic components.*

References added:

*57.) Monzón M, Ortega Z, Hernández A, Paz R, Ortega F. Anisotropy of Photopolymer Parts Made by Digital Light Processing. Materials. 2017;10. doi:10.3390/ma10010064*

*58.) Restrepo S, Ocampo S, Ramírez JA, Paucar C, García C. Mechanical properties of ceramic structures based on Triply Periodic Minimal Surface (TPMS) processed by 3D printing. Journal of Physics: Conference Series. 2017;935: 012036. doi:10.1088/1742-6596/935/1/012036*

*59.) Yao Y, Qin W, Xing B, Sha N, Jiao T, Zhao Z. High performance hydroxyapatite ceramics and a triply periodic minimum surface structure fabricated by digital light processing 3D printing. Journal of Advanced Ceramics. 2021;10: 39–48. doi:10.1007/s40145-020-0415-4*

*60.) Baino F, Magnaterra G, Fiume E, Schiavi A, Tofan L-P, Schwentenwein M, et al. Digital light processing stereolithography of hydroxyapatite scaffolds with bone-like architecture, permeability, and mechanical properties. Journal of the American Ceramic Society. 2022;105: 1648–1657. doi:10.1111/jace.17843*

Supporting Information added:

***S10 Fig. Flexural testing and proposed DLP designs to investigate bone formation.***

*(A) Illustrations of an example build plate for DLP 3D printing of LithaBone HA400 horizontal and vertical rupture test bars for a four-point bend flexural test set up, where flexural strength is measured for vertical (top) and horizontal (bottom) 3D printed bars. (B) Plot of flexural strength (MPa) versus open porosity (%) for fired (sintered) LithaBone HA400 modulus of rupture test bars, depend on their print orientation (horizontal vs vertical). (C) Clover and trifurcating LithaBone HA400 scaffold designs for proposed use in bone formation experimental studies. Images of scaffold were taken at 200x magnification using a Hirox 3D digital microscope and right most image is of a clover design LithaBone HA400 scaffold stained with nuclear fast (pink/red; this stain is part of the von Kossa staining protocol and it stains nuclei) to visualize presence of U2OS cells after 14 days of culture. Scale bar is 1mm .*

7.) Reviewer 2 comment “…One general criticism according to me is that the HA material itself is only very poorly detailed/characterized and the main results are focusing on cell response. Although cell response is indeed capital in this type of application, the characteristics of the biomaterial itself is also of prime importance. I have well noted that a commercial HA slurry was used, however a more detailed description of the HA characteristics would be quite necessary. At minimum, the XRD pattern, FTIR spectrum, particle size distribution, Ca/P ratio, eventual degree of carbonation etc... would be relevant to give to better apprehend the cell behavior results and their relevance to the field. “Minor impurities” are mentioned on page 12 but no more details are given. Again, which impurities, to which extent etc would help to strengthen the impact of the paper.

**Correction:** Reviewer 2 brings up a valid point regarding material characterization of LithaBone HA 400, however, due to the fact that the material is commercially available, and protected behind Lithoz patents and trademarks, we mirrored material analysis efforts similar to our listed reference 32 (Schmidleithner C, Malferrari S, Palgrave R, Bomze D, Schwentenwein M, Kalaskar DM. Application of high resolution DLP stereolithography for fabrication of tricalcium phosphate scaffolds for bone regeneration. Biomedical Materials. 2019;14: 045018. doi:10.1088/1748-605x/ab279d), which focused on material characterization and analysis relevant to biocompatibility. Similar to this published study, we also conducted SEM analysis (Fig 2) to understand material porosity and surface properties, X-ray diffraction (XRD) analysis (S2 Fig) to evaluate the crystalline phases of the material, this is also how we identified the presence of minor impurities (corrected to “minor secondary phases”) in the printed material. In addition, we also conducted surface roughness (S3 Fig) and hydrophobicity (S4 Fig) analysis of the sintered prints. Reviewer 1 has similar concerns and we have included changes to revised manuscript to address these points. Please see corrections already made in reference to reviewer 1 comments in our response #6 (see below):

(Lines 138-141) See our response (6) to reviewer 1’s concerns.

(Lines 352-354) *Fired coupons were analyzed using powder X-ray diffraction (XRD). The XRD pattern was consistent with hydroxyapatite (HA), demonstrating coupons to be predominately HA with very minor secondary phases as shown in S2 Fig.*

**Correction-continued:** In support of reviewer 2 point of concern regarding material characterization, we included additional characterization data (FTIR and ICP-OES), data shown in S2 Fig. We also further clarified that additional analysis is planned for a future study where the composition and makeup of the slurries used to DLP 3D print ceramics for use in orthopedic applications. The following changes were made:

(Lines 166-173) *Powder X-ray Diffraction (XRD) and Fourier-transform Infrared Spectroscopy (FTIR) was used to identify the crystalline phases of the fired coupons. Fired coupons were crushed into a powder and analyzed by a Bruker D8 Discover machine for XRD and with a Bruker Optics, Hyperion 3000 IR microscope attached to a Bruker Optics, Vertex 70 FTIR. For FTIR, the powder was pressed between diamond plates to obtain a thin film for the transmission measurement in the Hyperion 3000 IR microscope. The NIST standard spectrum for calcium phosphate hydroxide was used for comparative analysis (NIST website: https://webbook.nist.gov).*

(Lines 354-356) *The IR spectrum obtained by FTIR shows expected peaks for hydroxide (OH^-^) and phosphate (PO_4_)^-3^ as compared to the NIST standard spectrum for calcium phosphate hydroxide.*

(Lines 674-678) *Development of novel slurries for DLP printed materials for orthopedic and clinical applications would benefit from an in-depth material characterization analysis (i.e. FTIR spectrum, particle size distribution within slurries, Ca/P ratio and degree of carbonation sintered prints) to aid in apprehension of cell response to DLP produced ceramic components.*

(Lines 357-358) *ICP-OES was also conducted, and measured Ca/P ratios were on average 1.66, see S2 Fig, C.*

(Lines 178-193) *Inductively Coupled Plasma-Optical Emission Spectroscopy (ICP-OES) analysis was conducted on LithaBone HA400 powder. Samples were prepared for ICP-OES analysis by hot block digestion. All data was collected using a spectro ICP-OES instrument with a CETAC Autosampler ASX-520, Tracey Teflon cyclonic spray chamber, MiraMist nebulizer and D-Torch with ceramic outer tube and alumina inner tube, tapered alumina injector. Sample preparation was as follows: LithaBone HA400 powder was ground with a mortar and pestle and 0.05 g of powder was added to a pre-weighed 50 mL natural cap tube. To this, 4 mL of HNO_3_/HCl 25/75 vol% was added to the tube and the tubes were placed inside a heat block at 90 °C for 30 minutes to allow for digestion. After incubation, de-ionized water was added to the 50 mL fill line on the tubes and the tubes were re-weighed. A 100-fold dilution was made of the prepared digestion solution using 3% HCl, 1% HNO_3_, 0.5 %HF in 15ml natural cap tubes. Internal standard of Sc was added to matrix (1ml), followed by acid and filled with deionized water to the 10ml gradation mark. ICP-OES analysis results for Ca, P, and Ca/P ratios are shown in S2 Fig.*

Supporting Information revised:

***S2 Fig. Phase identification using X-ray diffraction, Fourier-transform Infrared Spectroscopy, and Inductively Coupled Plasma-Optical Emission Spectroscopy.***

*(A) The fired coupon was analyzed by X-ray diffraction (XRD) to evaluate the crystalline phases. The XRD pattern was consistent with hydroxyapatite and showed only three very minor secondary phase peaks, which were consistent with orthorhombic hydroxyapatite and magnesium oxide (arrows in inset). (B) The coupon was also analyzed by Fourier-transform Infrared Spectroscopy (FTIR), revealing the IR spectrum of LithaBone HA400 powder in diamond transmission cell. (C) Inductively Coupled Plasma-Optical Emission Spectroscopy (ICP-OES) was used to characterize the average calcium/phosphate (Ca/P) ratio of coupons. Analysis was done on fired (sintered) LithaBone HA400 powder (Sample IDs S22-01607a-d). Wt%, Mol%, and Ca/P ratio was measured.*

8.) Reviewer 2 comment: “…The terms ‘photocurable HA slurry’ are used but the curability is unclear. More details would be interesting so as to more clearly understand the type of interactions that may occur during processing (which resin? which mechanism of curing?...).

**Correction:** To reviewer 2 point of concern regarding the use of the term ‘photocurable HA slurry’ needed more context regarding its make-up to better understand processing, we have included the following changes in the revised manuscript:

(Lines 132-137) *Commercial LithaBone HA400 (Lithoz GmbH) slurry, consisting of hydroxyapatite dispersed in a photocurable resin (similar to slurries used in Schmidleithner, C. et al [33], hydroxyapatite (HA) power was suspended in an organic matrix consisting of acrylates and methacrylates with a radical photoinitiator absorbing in the blue visible region [34–36]), was used with the CeraFab 7500 (Lithoz GmbH) where selective exposure to visible light cures the photocurable slurry.*

References added:

*34.) de Blas Romero A, Pfaffinger M, Mitteramskogler G, Schwentenwein M, Jellinek C, Homa J, et al. Lithography-based additive manufacture of ceramic biodevices with design-controlled surface topographies. The International Journal of Advanced Manufacturing Technology. 2017;88: 1547–1555. doi:10.1007/s00170-016-8856-1*

*35.) Schwentenwein M, Schneider P, Homa J. Lithography-Based Ceramic Manufacturing: A Novel Technique for Additive Manufacturing of High-Performance Ceramics. Advances in Science and Technology. 2014;88: 60–64.*

*36.) Schwentenwein M, Homa J. Additive Manufacturing of Dense Alumina Ceramics. International Journal of Applied Ceramic Technology. 2015;12: 1–7. doi:https://doi.org/10.1111/ijac.12319*

9.) Reviewer 2 comment: “…The introduction section, comparisons are made with metal based systems. However, the relevance of metal-based protheses lies in their mechanical properties which is a point that does not seem to be considered, instead the authors mention the non resorbability of the metals, but this seems out of scope for metals. Please rethink this part.

**Correction:** We agree with reviewer 2 comment regarding the mechanical advantage of metal implants. As such, we have in provided reference to this aspect (see changes made to revised manuscript below). However, one of the larger disadvantages of metal implants is its limited ability for osteointegration and is a center point in the scope of this work, where an alternative material, hydroxyapatite, is explored for used in DLP 3D printing process. Additively printed metal implantable parts have limited resorption potential as compared to HA. We also expanded the discussion around resorption of HA as compared to other ceramic base materials, such as β-tricalcium phosphate.

(Lines 58-66) *For large repairs, such as in spinal reconstruction or hip replacements, titanium implants are used over autologous bone due to the complexity and magnitude of material needed [11] and for its particular ability for meeting load-bearing mechanical needs [12].* *Advances in selective laser melting (SLM) techniques have led to the creation of porous titanium implants, allowing for increased cellular integration and biocompatibility with the implant (while maintaining the favorable mechanical properties of titanium) previously noted only on the external surface of titanium implants, at the metal tissue layer interface [13–15].*

(Line 671-674) *These types of composite slurries would have base components with both rapid (β-tricalcium phosphate) and slower (HA) rates of resorption tailored toward supporting patient specific bone repair needs.*

Reference added:

*12.)* *Bandyopadhyay A, Ciliveri S, Bose S. Metal Additive Manufacturing for Load-Bearing Implants. Journal of the Indian Institute of Science. 2022. doi:10.1007/s41745-021-00281-x*

10.) Reviewer 2 comment: “…Some terms do not seem adequate, like “plastics” instead of “polymers”, or the term “host”, both in page 4. Please check also some English terminology/spelling as for “biologic” versus “biological” on page 17.”

**Correction:** We agree with reviewer 2 points of concerns. We have made the following changes to the revised manuscript:

(Lines 85-87) *3D printing offers the greatest design and overall application flexibility, accommodating a variety of materials (polymers, ceramics, metals, blends), while finely tuning print features and controlling mechanical properties using CAD [27].*

(Lines 53-55) *Despite autologous bone grafting being named the gold standard of care for bone defect management, it suffers from various problems including donor site morbidity and limited quantity of available bone [8,9].*

(Lines 80-81) *Common techniques include electrospinning, gas-foaming and 3D printing, each with their own advantages and disadvantages and material processing capabilities [25].*

(Lines 446-448) *MSCs cultured on coupons were observed to have a spindle morphology consistent with healthy undifferentiated MSCs, confirming biological compatibility of the DLP printed HA materials.*

11.) Reviewer 2 comment: “…On page 7 a heat treatment is mentioned at 1300°C. Again, this may be mirrored with the HA composition as nonstoichiometry and/or carbonation (or perhaps also the impurities that are mentioned once in the text) could lead to partial decomposition. This is also why better characterization/compositional details would be greatly appreciated.

**Correction:** We conducted additional characterization analysis of coupons after sintering (heat treatment at 1300°C). To address this concern, we have revised Fig 2 and made the following changes to the revised manuscript:

(Lines 391-394) *SEM image of coupon also shows densification of LithaBone HA400 particles (measured to be ~2-6 micron in green state—not sintered prints) after firing (heat treatment at 1300°C), see Fig 2, C-D.*

Figure revised:

***Fig 2. Scanning Electron Microscopy (SEM) of DLP print builds.***

*(A) Coupons built using DLP process were rinsed with a propylene carbonate/tripropylene glycol blend and then fired. (B) For SEM imaging, coupons were transversely sectioned mounted and polished, (C) revealing innate porosity. (D) SEM image of coupon before firing (green state, not sintered) reveals particle size of LithaBone HA400 ~2-6 microns and (E) after firing (heat treatment at 1300°C),densification of particles is seen as a result of sintering.*

12.) Reviewer 2 comment: “…To improve interactions with some cells, a “priming” with fibronectin or Matrigel is mentioned. But again, no chemistry data or quantification or proof of actual surface “functionalization” is shown to demonstrate the surface modifications undergone. Similarly, the “extractables” and “leachables” that are mentioned, e.g. on page 14, are only rapidly mentioned but deserve more attention. Which compounds are considered here? Did you run chemical analyses? Did you titrate chemically to determine the remaining amounts?”

**Correction:** Coating with fibronectin or Matrigel have been demonstrated to benefit cell attachment and our approach followed standard immersion procedures published in literature. We have added the following reference to the revised manuscript and made changes as follows:

(Lines 439-444) *In contrast, attachment efficiency of MSCs on HA coupons was reduced by 30% when compared to standard tissue culture polystyrene (S6 Fig, A). We speculated that pre-adsorption of extracellular matrix proteins onto the HA coupon would increase MSC attachment using a standard immersion procedure as used in Deligianni, et. al.[38]. The attachment efficiency of MSCs on coupons was greatly improved when coupons were coated with fibronectin or Matrigel (S6 Fig).*

Reference added:

*39.)* *Deligianni D, Korovessis P, Porte-Derrieu MC, Amedee J. Fibronectin preadsorbed on hydroxyapatite together with rough surface structure increases osteoblasts’ adhesion “in vitro”: the theoretical usefulness of fibronectin preadsorption on hydroxyapatite to increase permanent stability and longevity in spine implants. J Spinal Disord Tech. 2005;18: 257–262.*

**Correction-continued:** Regarding extractable and leachables, we assumed organic byproducts from the DLP 3D printing process, which may have remained after sintering my leach into the cell culture medium and affect cell proliferation or viability. Possible byproducts include organic compounds within the commercially obtained Lithobone HA400 slurry, which are volatile and are burned off during the sintering process. Our results demonstrate that the level of extractable and leachables of sintered Lithobone HA400 is insufficient to affect cell phenotype and their viability. We have clarified this point in the revised manuscript as follows:

(Lines 417-420) *To address potential leachable and extractable production during the DLP additive manufacturing process (e.g. impurities in the slurry, residual organic material from the printing process, byproducts of sintering, or inorganic contaminates), autoclaved coupons were exposed to cell culture medium for 3 days.*

13.) Reviewer 2 comment: “…Concerning the osteosarcoma cell line, perhaps few details stating which this could be a good cell model for this study could be worth adding.

**Correction:** The U2OS cell line represents a routine and well characterized cell type that mimics mesenchymal stem cells in size and attachment and growth characteristics. To address this concern, we have made the following changes to the revised manuscript:

(Lines 420-423) *U2OS human osteosarcoma cells, a routinely used and well characterized cell type that mimics mesenchymal stem cells in size and attachment and growth characteristics, were cultured in coupon conditioned medium for 48 hours.*

14.) Reviewer 2 comment: “…On page 17, the text right after the “Fig.5 title” seems to be the figure legend and not actual text.

**Correction:** We see the confusion identified by the reviewer. We have edited this section to add clarity and reduce duplication of language. To address this concern, we have made the following changes to the revised manuscript:

(Lines 484-491) *Similarly, MSC proliferation on HA DLP printed coupons was comparable to polystyrene (Fig 5). MSCs (1.0x10^4^ cells/cm^2^) were seeded at approximately 40% confluence onto tissue culture polystyrene 12-well plates and coupons and proliferation over 3 days was quantified using the RealTime-Glo^TM^ MT Cell Viability Assay (Promega). We observed that MSCs on uncoated HA coupons had a reduced attachment efficiency compared to coupons precoated with fibronectin or Matrigel (S6 Fig). Nonetheless, cell doubling time was similar for MSCs on HA coupons and standard tissue culture polystyrene plates at all seeding efficiencies (S7 Fig).*

15.) Reviewer 2 comment: “…On page 20, there is a mention of “TCP”. It is unclear which compound is considered here, please be more precise.”

**Correction:** To provide clarity, TCP is only used in reference to tissue culture polystyrene (TCP) and we have changed the mention of “β-TCP” to β-tricalcium phosphate in the revised manuscript. In addition, we have defined the mention of TCP on page 20 to say “tissue culture polystyrene” to improve clarity to readers. To address this concern, we have made the following changes to the revised manuscript:

(Lines 550-556) *MSCs seeded onto DLP printed coupons (two different batches: coupon A and coupon B and average “coupon”) or tissue culture polystyrene, “TCP” (12-well plates, TCP) were cultured in non-differentiation media or in osteogenic differentiation media and the (A) fold-increase in alkaline phosphatase activity on day 14 (n=3) for cells cultured in osteogenic differentiation media was measured and (B) cells were processed for mRNA extraction and analysed by RT-PCR for gene expression of Runx2, SP7, SPP1, and GAPDH on day 21 (n=2).*

(Lines 561-566) *A similar elevation in alkaline phosphatase activity and Runx2 mRNA expression as compared to GAPDH (control) (P>0.05) was observed for cells on coupons and on tissue culture polystyrene, “TCP”, indicating comparable osteogenic differentiation efficiency both early markers for osteogenic differentiation, but not for SP7 and SPP1 mRNA expression, both considered late-stage differentiation markers.*

(Lines 670-674) *Such efforts might include the integration of β-tricalcium phosphate and other resorbable elements into the slurry system [18]. These types of composite slurries would have base components with both rapid (β-tricalcium phosphate) and slower (HA) rates of resorption tailored toward supporting patient specific bone repair needs.*

Lines (968-969) *Coupons have ~30% fewer number of cells compared to tissue culture polystyrene, “TCP”.*

16.) Reviewer 2 comment: “…Finally, this 3D approach is notably pertinent if there is a need to make complex 3D shapes. It could have been a nice addition toward the end of the paper to show that this DLP approach can also lead to complex shapes… Also, some info about the limitations in the size of the 3D objects that can be prepared by this approach could have been interesting.”

**Correction:** With DLP we can 3D print features with 1 micron control and have included an additional figure in the supportive information showcasing examples of the various shapes (clover and trifurcating) we have created and are currently using in bone formation studies with academic collaborators. To address this concern, we have added S10 Fig in the supporting information and have made the following changes to the revised manuscript:

(Lines 638-646) *Future work will leverage these results toward design and cell performance on 3D scaffolds with complex internal structures that mimic trabecular bone; some examples are depicted in S10 Fig, C which represent unit cell structures we have successfully printed by DLP that we believe may have relevance for supporting bone regeneration. Within these examples, we can print features with micron control (~40µm resolution with minimum feature size ~150µm for complex parts) and regulate the local topologies experienced by seeded cells.* *Notably, designs can be tailored to have architectural elements promoting cellular integration and responsiveness [25].*

Supporting Information added:

***S10 Fig. Flexural testing and proposed DLP designs to investigate bone formation.***

17.) Additional edits were made to the manuscript and were as follows:

**Correction:** Addition of Inductively Coupled Plasma - Optical Emission Spectroscopy (ICP-OES) analysis in S2 Fig was included, which measured average Wt%, Mol%, and Ca/P ratios of LithaBone HA400. We have made the following changes to the revised manuscript as follows:

Supporting Information revised:

***S2 Fig. Phase identification using X-ray diffraction, Fourier-transform Infrared Spectroscopy, and Inductively Coupled Plasma-Optical Emission Spectroscopy.***

**Correction-continued:** For consistency and clarity, changes to the revised manuscript were made as follows:

(Lines 39-40) *We conclude that the DLP process and LithaBone HA400 slurry are biocompatible and are suitable for osteogenic applications.*

(Lines 541-545) *In all cases, the osteogenic differentiation increased in differentiation culture conditions (Fig 6 and Fig 7, A), with increased expression of differentiation markers when compared to GAPDH (control) (Fig 7, B) and positively correlated with staining of Alizarin Red S (Fig 6, Fig 7, A and S8 Fig.) or von Kossa (Fig 6 and S9 Fig.).*

(Lines 609-611) *Interestingly, MSCs in osteogenic differentiation medium reorganized on both polystyrene plates and HA coupons, resulting in cell morphology contraction, but this did not negatively impact osteogenic differentiation (Fig 6, Fig 7, S8 and S9 Fig).*

(Lines 350-351) *Phase identification, density, characterization, and contact angle measurements.*

(Lines 155-157) *The first step was a low temperature firing to 205 °C using a DKN602C oven (Yamato)* *to remove volatile organic components prior to high temperature sintering.*

(Line 85-87) *3D printing offers the greatest design and overall application flexibility, accommodating a variety of materials (polymers, ceramics, metals, blends), while finely tuning print features and controlling mechanical properties using CAD [27].*

(Lines 151-152) *Print layer thickness of 25 microns was selected and the cure time per layer was 3.5 seconds at full LED intensity (56 mW/cm^2^).*

(Lines 176-177) *The sample was mounted on the universal sample stage, set in the reflection geometry, and oscillated along the X and Y directions.*

(Lines 468-471) *Using this assay, U2OS cells seeded at roughly ~25% (1.75x10^3^ cells/cm^2^) confluence in 12-well tissue culture plates and on HA DLP printed coupons were observed to have comparable proliferation rates (P>0.5; using T-test, differences were not statistically significant) (Fig 4).*

(Lines 509-513) *While calcium deposition assays were complicated by the presence of calcium phosphate in HA coupons and the opacity of the coupons, the increased frequency and size of gray/black punctate staining for von Kossa and dark red for Alizarin Red S, indicates cellular calcium deposition on DLP printed coupons.*

(Lines 68-70) *Titanium implants have also been shown, in some cases, to trigger an immune response (particularly in dental implants), [17,19] and concern over heavy metal toxicity [20] have also been raised.*

(Lines 89-92) *For example, synthetic (i.e. poly(lactic acid) (PLA)) and natural (i.e. hyaluronic acid (HA)) polymers [29], proteins [27,28,30], and inorganic materials (i.e. hydroxyapatite) [31] have all been widely characterized for bone regeneration applications.*

(Lines 108-110) *In this, paper, we confirm that the DLP process can fabricate biocompatible hydroxyapatite structures that support osteogenesis.*

(Lines 118-123) *Using the DLP printing system (outlined in Fig 1), a modified circular coupon geometry (17.5mm length, 21mm diameter, and 0.5mm thickness) (see Fig 2, A for image of coupon after printing and S1 Fig for illustrations of coupon dimensions and print direction) was selected to maximize the coverage area of a single cell in a standard 12 well plate and provide feasibility for material characterization and biocompatibility.*

(Lines 207-208) *Samples were processed and roughness analyzed using the standard Keyence software for the profilometer, see S3 Fig.*

(Lines 319-338) ***Simple geometrical 3D DLP printed, coupon design.****Coupons in this paper were 3D printed using the DLP manufacturing process, illustration shown in Fig 1, utilized a commercial LithaBone HA400 (Lithoz GmbH) slurry. Coupons were printed with simple geometry, designed for effective handling in single wells of tissue culture polystyrene (TCP) plates (12-well) and evaluation of the coupon material for the potential use of the DLP process in future development of more complex geometries. LithaBone HA400 slurry poured into a vat and light, projected from below and onto the build plate, cures, one layer at a time, each layer 25µm thick. The coupon prints were built to have 17.5mm length, 21mm diameter, 0.5mm thickness. Coupons, circular in shape, were also designed with a hole on the edge to facilitate handling with sterile forceps (S1 Fig, A), which facilitated transfer of coupons in and out of single wells within TCP plates. Coupon geometry was also designed with a flat edge, where coupon print is adhered to the build plate and the layers grow outward in the direction of the arrow shown in S1 Fig, B. This geometrical design proved effective for minimizing print failures and damage during removal from build plates, all while maximizing the available space for printing coupons on a single build plate. Printed coupons were cleaned, then heated to remove the light curable resin and sinter the ceramic. The simple coupon design presented in this paper, allowed for ease of material characterization and evaluation of biocompatibility of hydroxyapatite DLP printed material. Use of simple coupon designs are commonly employed for evaluation of potential orthopaedic materials.*

(Lines 377-380) *Light profilometry was used to measure surface roughness of DLP printed coupons. An example roughness profile of an HA coupon is shown in S3 Fig for both parallel (vertical) and perpendicular (horizontal) directions with respect to the build direction.*

(Lines 513-515) *MSCs cultured in standard growth media did not show similar punctate calcium staining with either alizarin Red S (S8 Fig) or von Kossa (S9 Fig).*

(Lines 527-546) *In addition, MSCs cultured in osteogenic medium for 14 days exhibited 3.5-4 folder higher (P<0.05) alkaline phosphatase activity than MSCs in MSC growth medium on both DLP printed HA coupons and standard polystyrene tissue culture plates (Fig 7, A). Similar fold increase was observed for mRNA expression of Runx2 as compared to GAPDH (control) (P<0.05), an early stage osteogenic differentiation marker [40] for MSCs cultured in osteogenic medium for 21 days, but not for SP7 and SPP1, both considered late-stage osteogenic differentiation markers [41–44] (Fig 7, B). The post-differentiation increase in Runx2 mRNA expression was higher for MSC differentiated on HA coupons as compared with MSC differentiated on standard polystyrene tissue culture plates, while the expression of osterix (SP7), and osteopontin (SPP1), was lower for MSC differentiated on HA coupons. Thus, the material itself may influence the kinetics of differentiation. This pattern of expression resembles an in vivo-like response, where Runx2, an early osteogenic differentiation marker [40,45], peaks near day 21 as compared to SP7 [41,42], and SPP1 [46,47], both later differentiation markers.* *In all cases, the osteogenic differentiation increased in differentiation culture conditions (Fig 6 and Fig 7, A), with increased expression of differentiation markers when compared to GAPDH (control) (Fig 7, B) and positively correlated with staining of Alizarin Red S (Fig 6, Fig 7, A and S8 Fig.) or von Kossa (Fig 6 and S9 Fig.). These data confirm MSC osteogenic differentiation is supported on DLP printed HA coupons.*

Figures revised:

***Fig 1. Illustration of DLP process.***

*(A) The DLP process starts with a light curable resin and a powder, which are combined to make a photocurable slurry. We utilized a premixed hydroxyapatite photocurable slurry. (B) Slurry is poured into the vat where the (C) light is projected from below onto the build plate, curing one layer at a time of the part (e.g., coupons). (D) The printed part (coupon) is removed from the build plate and cleaned, then placed in a furnace where the light curable resin is burned out and the ceramic densifies, (E) creating the final part (coupon).*

***Fig 2. Scanning Electron Microscopy (SEM) of DLP print builds.***

*(A) Macro view of coupons built using DLP process, which were rinsed with a propylene carbonate/tripropylene glycol blend and then fired. Micro view and analysis of coupons using SEM imaging was conducted on coupons (B) transversely sectioned mounted and polished, (C) revealing innate porosity. (D)* *SEM image of coupon before firing (green state, not sintered) reveals particle size of LithaBone HA400 ~2-6 microns and (E) after firing (heat treatment at 1300°C),densification of particles is seen as a result of sintering.*

*(Updated Image)* ***Fig 3. MSCs morphology on DLP printed coupons.***

***Fig 4. U2OS proliferation on DLP printed HA coupons.***

*Relative luminescence unit (RLUs) were measured from U2OS cells seeded at ~25% (1.75x10^3^ cells/cm^2^) density on polystyrene plates or DLP printed HA coupons using RealTime-Glo^TM^ MT Cell Viability Assay. Cell counts were enumerated based on RLU measurements for the 4 and 48 hour time points using a standard curve correlating cell count and RLU (S7 Fig). Direct cell count was done at 66 hours of incubation by image analysis (counted cell nuclei). U2OS cells proliferation rates were similar on coupons and tissue culture polystyrene plates (P>0.5; using T-test, differences were not statistically significant).*

*(Updated Image)* ***Fig 5. MSC proliferation on DLP printed coupons.***

***Fig 6. Calcium deposition from osteogenic differentiated MSCs on DLP printed coupons.***

*(A) Calcium deposits stain red when cells are stained with Alizarin Red S and (B) gray to black with von Kossa. (B) The von Kossa counter stain, nuclear fast red, stains cells a fuchsia color. The observation of punctate staining in MSCs cultured in osteogenic differentiation medium on DLP printed coupons and polystyrene plates demonstrates calcium deposition on both surfaces using both Alizarin Red S and von Kossa staining. In contrast, MSCs cultured in growth media lack similar punctate calcium staining with either Alizarin Red S or von Kossa. Images were taken using Lecia dissection scope at 4x magnification, scale bar 1.25mm.*

***Fig 7. Expression of alkaline phosphatase and RT-PCR analysis for MSCs differentiated on DLP printed HA coupons.***

*MSCs seeded onto DLP printed coupons (two different batches: coupon A and coupon B and average “coupon”) or tissue culture polystyrene, “TCP” (12-well plates, TCP) were cultured in non-differentiation media or in osteogenic differentiation media and the (A) fold-increase in alkaline phosphatase activity on day 14 (n=3) for cells cultured in osteogenic differentiation media was measured and (B) cells were processed for mRNA extraction and analysed by RT-PCR for gene expression of Runx2, SP7, SPP1, and GAPDH on day 21 (n=2). MSCs were cultured either in osteogenic differentiation media or non-differentiation media. Ratio of differentiation for alkaline phosphatase was calculated by measuring absorbance at OD 405nm. Post-differentiation fold increases in mRNA expression of targeted genes were calculated by measuring RT-PCR ct values of cells cultured in osteogenic differentiation media over that of cells in non-differentiation media.* *A similar elevation in alkaline phosphatase activity and Runx2 mRNA expression as compared to GAPDH (control) (P>0.05) was observed for cells on coupons and on tissue culture polystyrene, “TCP”, indicating comparable osteogenic differentiation efficiency both early markers for osteogenic differentiation, but not for SP7 and SPP1 mRNA expression, both considered late-stage differentiation markers.*

Supporting Information revised:

***S4 Fig. Contact angle approach to measure hydrophilicity of coupons.***

*(A) Pictures of water droplet on coupon surface, image taken 2 seconds after liquid contacted the surface of the coupon, resulting in a contact angle of (L,R) 73.5°, 74.5° and (B) a contact angle of (L,R) 0°, 0° with the water fully spread across coupon surface. Images demonstrate the contact angle approach used to measure hydrophilicity of coupons.*

***S5 Fig. Extractable and leachables study using U2OS cells.***

*Seeded at ~66% confluency (4.6x10^3^ cells/cm^2^), U2OS cells were exposed to coupon conditioned culture media for 48 hours. Cells cultured on tissue culture polystyrene (TCP) and in the presence of coupon conditioned culture media were observed to maintain high confluency with no negative impact on morphology or proliferative capabilities. Image was taken using EVOS, phase contrast, 10x objective magnification, scale bar is 250µm.*

*(Updated Image)* ***S6 Fig. Uncoated DLP printed coupons have reduced cell attachment efficiency.***

We look forward to your response regarding our revised manuscript for publication in PLOS ONE.

Sincerely,

Dr. Jessica Martinez

Lead Scientist; Biosciences

GE Research

One Research Circle

Niskayuna, NY 12309

Office: 518-387-4506

Mobile: 518-386-9566

E-mail: [jessica.s.martinez@ge.com](mailto:jessica.s.martinez@ge.com)
